# Prevalence and co-occurrence of modifiable risk factors in adults and older people

**DOI:** 10.11606/s1518-8787.2019053001142

**Published:** 2019-10-16

**Authors:** Priscila Maria Stolses Bergamo Francisco, Daniela de Assumpção, Flávia Silva Arbex Borim, Caroline Senicato, Deborah Carvalho Malta

**Affiliations:** I Universidade Estadual de Campinas. Faculdade de Ciências Médicas. Departamento de Saúde Coletiva. Campinas, SP, Brasil; II Universidade Federal de Minas Gerais. Escola de Enfermagem. Belo Horizonte, MG, Brasil

**Keywords:** Adult, Aged, Noncommunicable Diseases, prevention & control, Risk Factors, Sedentary Behavior, Healthy Lifestyle, Health Knowledge, Attitudes, Practice

## Abstract

**OBJECTIVE:**

To estimate the co-occurrence of the major risk factors for chronic diseases in adults (18-59 years old) and older people (≥ 60 years old) living in Brazilian state capitals and the Federal District.

**METHODS:**

Cross-sectional study with population-based data from 35,448 adults and 18,726 older people collected in the *Sistema de Vigilância de Fatores de Risco e Proteção para Doenças Crônicas por Inquérito Telefônico* (System of Surveillance of Risk and Protective Factors for Chronic Diseases by Telephone Survey – Vigitel) in 2015. The prevalence of each of the five risk factors (smoking, overweight, physical inactivity, alcohol and unhealthy diet) was estimated, as well as their co-occurrence for the different possible combinations, according to socioeconomic and health self-assessment variables. The independent associations were verified via multinomial logistic regression to obtain the estimates of the odds ratio (OR) and corresponding 95% confidence intervals.

**RESULTS:**

At least two risk factors were present in 38.5% of the adults and 37.0% of the older participants. The male adults and older participants who did not have private health insurance and classified their health as average or poor/very poor were more likely to have two or more concurrent risk behaviors. The greater chance of co-occurrence of smoking and alcohol abuse in adults (adjusted OR = 3.52) and older people (adjusted OR = 2.94) stands out.

**CONCLUSIONS:**

The subgroups with increased risk of developing multiple unhealthy behaviors and the most prevalent behaviors were identified. These findings are expected to contribute to the better targeting of health promotion and preventive care. It is worth noting that, for the adoption of healthy lifestyle habits, macro-social and inter-sectoral policies are more effective.

## INTRODUCTION

Modifiable risk factors are harmful actions that increase the probability of occurrence of the disease or prevent the recovery of health^1^ . They are components of the causes of diseases and health conditions, with impact on the incidence of the morbidity and mortality associated with noncommunicable chronic diseases (NCD) – especially heart diseases, diabetes mellitus and cancer – in adults and older people^2^ .

According to the World Health Organization (WHO), a small set of risk factors is responsible for most deaths from NCD and for a significant proportion of the disease burden attributed to them^6^ . Smoking, alcohol abuse, physical inactivity and unhealthy diet are the main risk factors related to the morbidity and mortality associated with NCD ^6 , 7^ . Behavioral risk factors cause metabolic changes, such as excess weight (overweight or obesity), accounting for 5.0% of all deaths caused by NCD in the world^6^ . Several epidemiological studies have shown the contribution of these factors in determining the diseases^5 , 8 , 10^ .

The simultaneous presence of two or more risk factors enhances the chance of occurrence of NCD^2 , 11^ , and is associated with total mortality and mortality attributed to specific causes^3 , 14^ in men and women^14^ . In general, the risk factors related to lifestyle do not occur in isolation among individuals, but in groups, and are not distributed randomly across the population^15^ .

In a study on the effect of potentially modifiable risk factors associated with myocardial infarction in 52 countries, tobacco use, unhealthy diet, hypertension, diabetes mellitus and psychosocial stress accounted for 90% and 94% of the attributable risk for heart disease among men and women, respectively^11^ . In Brazil, a study that estimated the attributable risk fraction for 25 types of cancer caused by exposure to several modifiable risk factors (smoking, alcohol consumption, diet, overweight and obesity, physical inactivity, occupational and environmental agents, among others) concluded they will account for 34% of cancer cases among men and 35% among women in 2020, and for 46% and 39% of deaths, respectively^10^ .

National studies have been mainly considering smoking, unhealthy diet, physical inactivity, alcohol consumption and overweight in the analysis of accumulation of risk factors in the population. In adults, the occurrence of two or more factors was higher in men and in the segments of lower per capita income and education, having also decreased with age in a study conducted by Silva et al.^16^ ; in the older population, it was smaller the higher the individual’s age^13^ . Among the mentioned factors, the improper diet marker that is generally used is insufficient consumption of fruits and vegetables, reflecting a lack of more comprehensive information about the food profile.

Access to private health plans is rarely included in the assessment of co-occurrence of risk factors. This indicator can be considered to distinguish social strata^17^ , aiming at the implementation of public health promotion and disease prevention policies that consider specific subgroups. The relationship between the self-assessment of health – which integrates the individual’s biological, psychological and social perception – and the co-occurrence of risk factors is also not well known. The simultaneous prevalence of the major risk factors for NCD and their distribution in the adult and older population is still poorly dimensioned. Exposure to behavioral risk starts early^12^ , becoming consolidated in adulthood^16^ , and has negative effects on health in the various stages of life. In addition to the potential years of life lost to death or disability at earlier ages, the impact of exposure to these factors throughout life among individuals at more advanced ages should be considered. In both age groups, these conditions represent an important demand for health services, as well as family and social support. Promotion and prevention measures are more effective, depending on the adoption of a specialized approach to age groups and socio-demographic characteristics.

Assuming that there is a high prevalence of accumulation of risk factors in the population and that their occurrence varies according to socio-demographic characteristics and perceived health, the goal of the study was to estimate the prevalence of co-occurrence of the major risk factors for NCD in adults and older people in the Brazilian state capitals and the Federal District in 2015, and its association with sociodemographic characteristics and self-assessment of health.

## METHODS

This was a cross-sectional study conducted with adults (18 to 59 years old) and older people (≥ 60 years old) living in the Brazilian state capitals and the Federal District (FD). Data were obtained from a random sample of residents of households with at least one landline (n = 54,174), and collected by the *Sistema de Vigilância de Fatores de Risco e Proteção para Doenças Crônicas por Inquérito Telefônico* (System of Surveillance of Risk and Protective Factors for Chronic Diseases by Telephone Survey – Vigitel) in 2015.

Vigitel defines a minimum size of approximately 2,000 individuals in the sample of each city to estimate, with 95% confidence level and 2% maximum error, the frequency of the major risk factors for noncommunicable chronic diseases^18^ . In the first sampling stage, zip code numbers taken from telephone records of companies serving the 26 capitals and the FD were systematically drawn. After the lines eligible for the system had been identified, one of the adults (≥ 18 years old) living in the selected household was randomly selected. In 2015, Vigitel identified 76,703 eligible lines, obtaining 54,174 interviews^7^ .

The weights assigned to the individuals selected consider two factors: the inverse of the number of landlines in the household, which corrects the higher chance that individuals from households with more than one line had of being selected, and the number of adults in the respondent’s household, which corrects the lower chance that individuals living with other people had of being selected. The product of these factors provides a sample weight that allows calculating reliable estimates for the adult population with a landline in each city^7^ .

Finally, the post-stratification weight was applied, based on 36 categories of analysis by sex, age and educational level, allowing to match the sociodemographic composition estimated for the adult population with a landline in each capital to the total sociodemographic composition estimated for the adult population^7^ .

### Risk Factors

The risk factors analyzed were selected based on their importance for the determination of the total disease load estimated by WHO for the region of the Americas^20^ . In addition to them, an unhealthy diet indicator was created, considering a set of foods related to the prevention and risk of NCD.

### Smoking

Expressed by the percentage of smokers among the individuals interviewed. Smokers were defined as those who answered positively to the question “Do you smoke?”, regardless of the number of cigarettes or for how long they had smoked.

### Overweight or Obesity

Percentage of subjects with body mass index (BMI) ≥ 25 kg/m^2 , 21^ , obtained by dividing weight (kg) by the square of height in meters, both self-reported. When respondents were unaware of their weight or height, the values of these measures were imputed using the hot deck technique^7^ .

### Physical Inactivity

Percentage of individuals who did not practice any physical activity during leisure time in the last three months and whose work did not require intense physical exertion, who did not commute to the place of work or study by walking or cycling for at least 20 minutes, or who did not perform a deep cleaning of their home^7^ .

### Abusive Consumption of Alcohol

Percentage of individuals who consumed alcohol abusively (five or more drinks for men and four or more drinks for women) at least once in the last 30 days. In Vigitel, a dose of beverage corresponds to a can of beer, a glass of wine or a shot of liquor, whiskey or other alcoholic distilled drink.

### Unhealthy Eating

The unhealthy diet indicator was based on a set of foods considered to support the prevention of chronic diseases (fruits, raw and cooked vegetables, milk and beans) or increase their risk (sweets, red meat, soft drinks and other sweetened beverages). Points were assigned considering the frequency of consumption and the types of foods, on a scale ranging from zero to four. The score was calculated in reverse, i.e., the minimum score (zero) was attributed to the daily consumption of foods that support prevention and to the rare or absent consumption of foods that increase risk. The maximum score (four points) was assigned to the rare or absent consumption of foods that support prevention and to the daily consumption of foods that increase risk ( Table 1 ). The total score comprised the sum of the food items, ranging from 0 (best) to 32 points (worst quality of diet). The total score was categorized into terciles of the distribution, and then, individuals belonging to the 2nd and 3rd tercile (≥ 13 points) were grouped together to compose a dichotomous variable for unhealthy diet (yes or no).

**Table 1:** Score for the consumption of food in an unhealthy way. Vigitel, Brazil, 2015.

Foods	0	1	2	3	4
Beans	Daily	5 to 6 days a week	3 to 4 days a week	1 to 2 days a week	Never or hardly ever
Fruits	Daily	5 to 6 days a week	3 to 4 days a week	1 to 2 days a week	Never or hardly ever
Raw vegetables^a^	Daily	5 to 6 days a week	3 to 4 days a week	1 to 2 days a week	Never or hardly ever
Cooked vegetables^a^	Daily	5 to 6 days a week	3 to 4 days a week	1 to 2 days a week	Never or hardly ever
Milk	Daily	5 to 6 days a week	3 to 4 days a week	1 to 2 days a week	Never or hardly ever
Red meat^c^	Never or hardly ever	1 to 2 days a week	3 to 4 days a week	5 to 6 days a week	Daily
Soda or artificial juice	Never or hardly ever	1 to 2 days a week	3 to 4 days a week	5 to 6 days a week	Daily
Sweets^d^	Never or hardly ever	1 to 2 days a week	3 to 4 days a week	5 to 6 days a week	Daily

^a^ Lettuce and tomato salad or other raw vegetable salad.

^b^ Consumption of cooked vegetables with meals or in soup, like cabbage, carrot, chayote, eggplant and squash, excluding potato, yam, or cassava.

^c^ Beef, pork or goat meat.

^d^ Ice creams, chocolates, cakes, cookies and others.

### Statistical Analyses

The sociodemographic variables were: geographic macro-region of the country (North, Northeast, Midwest, South and Southeast), sex (male and female), skin color/ethnicity (white, mixed race, black and others), marital status (spouse, no spouse), education level (0-8, 9-11 and ≥12 years of study), occupational activity in the past three months (yes and no) and health insurance (yes and no). Self-assessment of health was also considered (very good or good, average and poor or very poor). The risk factors were encoded as binary variables (presence = 1 and absence = 0). From the sum of the individual behaviors, a score ranging from 0 to 5 was generated, based on the distribution observed. The created variable, number of risk factors, was categorized as none, one, two, three and four or more.

Initially, the prevalence of the number of factors was estimated according to the sociodemographic variables and to the self-assessment of health for both groups. The associations were verified by the crude odds ratios (OR) and 95% confidence intervals (95%CI) – multinomial logistic regression; reference category: absence of risk factors.

Subsequently, multiple analysis was conducted by selecting the number of factors (none, one, two, three or more) as dependent (polytomous) variable to obtain the adjusted estimates of the OR and respective 95%CI. All variables with association in the simple analysis (p < 0.20) were included in the initial model, and those with p < 0.05 remained in the final model. In this analysis, both groups were compared to the reference category (none of the five risk factors).

This study also estimated the co-occurrence of the prevalence of two factors (10 possible combinations, from the five factors evaluated). The odds ratios (OR) were obtained using binary logistic regression, expressed by the equation:

$$OR\;=\;\frac{{𝑓}_{11}\;𝒙{𝑓}_{00}}{{𝑓}_{10}\;𝒙\;{𝑓}_{01}\;}$$

where *f*_11_ is the number of individuals who reported both factors, *f*_00_ is the number of respondents who did not have any, *f*_10_ is the number of those in which the first factor was present, and *f*_01_ is the number of participants who reported the presence of the other factor only. In this way, a dichotomous variable was created, the category of interest of which was the presence of both risk factors (yes or no). The analyses were performed using Stata 14.0, considering the complex sample design.

The objectives of the survey were presented to the individuals by telephone, and the informed consent form was replaced by verbal consent. The study was approved by the National Ethics Committee for Research Involving Human Beings of the Brazilian Ministry of Health, under opinion no. 355,590, of June 26, 2013.

## RESULTS

The mean age was 40.0 years old (SD = 12.1) among the adults and 70.3 years old (SD = 7.9) among the older participants, with a higher percentage of women (52.6% and 60.7%, respectively). Of the adult population, 51.0% declared themselves as black or mixed, and of the older population, 33.8%. The proportion of subjects without a partner was higher among the adults (53.1%) than among the older participants (42.9%). Lower education level was observed among the older participants for all categories evaluated; while about 30.0% of the adults reported education ≥ 12 years, this percentage was only 17.0% for the older population. As for the subjective evaluation of health, 67.3% of the adults and 53.8% of the older participants assessed their health as very good or good, 28.4% and 38.8% as average, and 4.3% and 7.4 % as bad or very bad, respectively. Except for health insurance, in other variables, significant differences between adults and older people were observed (p < 0.05). Differences were also found in the assessment of individual risk factors. Adults had higher prevalence of smoking, alcohol abuse and unhealthy diet (p < 0.001) (data not shown).

A higher percentage of subjects with at least two risk factors (38.5%) was observed. Two risk behaviors were observed in 40.0% of the men, as well as high chances of occurrence of three (OR = 4.54, 95%CI 3.63–5.67) and four or more factors (OR = 5.93, 95%CI 4.16–8.45). The co-occurrence of two or more risk factors was higher in those with lower education level and in those who did not have health insurance at the time of the research. Lower prevalence of three and four or more risk factors was found among adults who were not working. Regarding the subjective evaluation of health, a higher prevalence of accumulation of risk factors was found among those who considered their health as average and poor or very poor ( Table 1 ).

**Table 1:** Prevalence and univariate analysis of the association between the number of risk factors for NCD, sociodemographic variables and self-assessment of health in the adult population. Vigitel, Brazil, 2015.

Variable	One		Two		Three		Four or more	
			
	n (%)	OR (95%CI)	n (%)	OR (95%CI)	n (%)	OR (95%CI)	n (%)	OR (95%CI)
Total	11,062 (34.4)		11,608 (38.5)		4,362 (15.5)		849 (3.3)	
Geographical region								
Southeast	1,495 (34.0)	1	1,554 (38.4)	1	584 (16.0)	1	134 (3.5)	1
Northeast	3,711 (35.1)	0.97 (0.80–1.18)	3,847 (39.0)	0.95 (0.78–1.16)	1,417 (14.7)	0.86 (0.69–1.09)	259 (2.7)	0.73 (0.51–1.03)
North	3284 (34.4)	1.16 (0.93–1.44)	3,438 (40.3)	1.20 (0.97–1.50)	1,259 (15.2)	1.09 (0.85–1.41)	234 (3.1)	0.99 (0.62–1.59)
Midwest	1,468 (34.5)	0.81 (0.61–1.08)	1,537 (36.6)	0.76 (0.57–1.02)	629 (15.3)	0.77 (0.56–1.06)	131 (3.6)	0.80 (0.50–1.30)
South	1,104 (34.1)	0.93 (0.73–1.17)	1,232 (38.4)	0.93 (0.73–1.17)	473 (15.8)	0.92 (0.70–1.20)	91 (3.2)	0.83 (1.55–1.25)
Sex								
Female	7,423 (40.7)	1	6,387 (37.2)	1	1,582 (9.5)	1	245 (1.7)	1
Male	3,639 (27.7)	**1.34 (1.10–1.63)**	5,221 (40.0)	**2.12 (1.74–2.58)**	2,780 (21.8)	**4.54 (3.63–5.67)**	604 (5.0)	**5.93 (4.16–8.45)**
Ethnicity/skin color								
White	4,313 (34.7)	1	4,469 (38.4)	1	1,701 (14.6)	1	387 (3.7)	1
Mixed race	4,549 (34.7)	1.11 (0.92–1.33)	4,736 (40.1)	1.16 (0.96–1.39)	1,746 (14.9)	1.13 (0.91–1.41)	300 (2.6)	0.76 (0.54–1.08)
Black	773 (35.4)	1.13 (0.78–1.64)	811 (37.2)	1.07 (0.74–1.56)	318 (16.0)	1.22 (0.80–1.85)	59 (3.6)	1.07 (0.58–2.00)
Other	490 (32.8)	0.70 (0.46–1.06)	514 (33.5)	**0.65 (0.43–0.98)**	174 (19.0)	0.97 (0.59–1.57)	26 (3.1)	0.62 (0.25–1.58)
Marital status								
Spouse	5,286 (32.8)	1	6,101 (40.2)	1	2,263 (15.6)	1	399 (3.3)	1
No spouse	5,562 (35.7)	1.05 (0.89–1.25)	5,330 (37.2)	0.89 (0.75–1.06)	2,043 (15.5)	0.96 (0.79–1.18)	434 (3.1)	0.91 (0.66–1.24)
Education level (years)								
≥ 12	4,953 (36.4)	1	4,915 (37.9)	1	1,838 (14.1)	1	349 (2.6)	1
9 to 11	4,524 (35.5)	1.04 (0.88–1.23)	4,724 (37.6)	1.06 (0.89–1.25)	1,779 (15.3)	1.15 (0.95–1.40)	332 (3.2)	1.30 (0.94–1.81)
0 to 8	1,585 (30.1)	1.00 (0.77–1.30)	1,969 (40.9)	**1.31 (1.01–1.70)**	745 (17.5)	**1.50 (1.11–2.03)**	168 (4.1)	**1.90 (1.25–2.89)**
Work in the last three months								
Yes	7,658 (33.6)	1	8,246 (38.7)	1	3,265 (16.4)	1	622 (3.4)	1
No	3,404 (36.5)	0.93 (0.77–1.12)	3,362 (38.2)	0.84 (0.70–1.01)	1,097 (13.2)	**0.69 (0.55–0.86)**	227 (2.8)	**0.70 (0.49–0.99)**
Private health insurance plan								
Yes	6,283 (36.3)	1	6,357 (36.5)	1	2,434 (14.9)	1	461 (2.9)	1
No	4,764 (32.4)	1.08 (0.99–1.18)	5,224 (40.6)	**1.21 (1.11–1.31)**	1,920 (16.1)	**1.19 (1.08–1.32)**	388 (3.7)	**1.28 (1.10–1.50)**
Self-assessment of health								
Very @good/good	8,052 (36.4)	1	7,651 (37.7)	1	2,693 (13.2)	1	492 (2.9)	1
Average	2,651 (31.2)	**1.59 (1.30–1.95)**	3,380 (39.7)	**1.95 (1.60–2.39)**	1,424 (20.3)	**2.86 (2.27–3.60)**	295 (3.5)	**2.20 (1.59–3.06)**
Poor/very poor	290 (23.4)	**2.22 (1.28–3.88)**	507 (46.2)	**4.24 (2.45–7.35)**	214 (20.2)	**5.30 (2.69–10.43)**	52 (7.4)	**8.72 (3.93–19.35)**

In bold: statistically significant associations.

In the older participants, the co-occurrence of three risk factors was higher among those living in the North region, and for four or more factors, it was lower in the Northeast, in relation to the Southeast of the country. The differences by sex remained, with higher prevalence among men. The co-occurrence of three or more risk factors was higher in mixed race individuals, compared to white individuals. It was also higher in those who did not exercise any occupational activity, who had no health insurance and who assessed their health as average and poor or very poor. Lower prevalence of two factors was observed in those living without a partner (OR = 0.73, 95%CI 0.57–0.93), and in those with up to 11 years of study, the co-occurrence of two or three factors was significantly lower ( Table 2 ).

**Table 2:** Prevalence and univariate analysis of the association between the number of risk factors for NCD, sociodemographic variables and self-assessment of health in the older population. Vigitel, Brazil, 2015.

Variables	One		Two		Three		Four or more	
			
	n (%)	OR (95%CI)	n (%)	OR (95%CI)	n (%)	OR (95%CI)	n (%)	OR (95%CI)
Total	5,432 (34.7)		5,318 (37.0)		2,046 (14.7)		255 (2.1)	
Geographical region								
Southeast	997 (34.9)	1	931 (37.0)	1	336 (14.2)	1	59 (2.5)	1
Northeast	1,546 (34.6)	0.99 (0.77–1.27)	1,533 (36.6)	0.98 (0.76–1.27)	622 (16.0)	1.12 (0.83–1.52)	62 (1.3)	**0.54 (0.30–0.97)**
North	1,051 (33.3)	1.26 (0.90–1.78)	1,047 (38.3)	1.37 (0.97–1.93)	391 (16.8)	**1.57 (1.06–2.32)**	57 (2.8)	1.49 (0.71–3.13)
Midwest	1,060 (35.0)	0.85 (0.64–1.14)	1,001 (35.2)	0.81 (0.60–1.08)	397 (14.4)	0.86 (0.61–1.21)	44 (1.9)	0.63 (0.33–1.23)
South	778 (34.8)	1.03 (0.77–1.37)	806 (38.7)	1.08 (0.81–1.44)	300 (13.8)	1.00 (0.71–1.41)	33 (1.6)	0.67 (0.35–1.29)
Sex								
Female	3,693 (38.5)	1	3,333 (35.6)	1	1,157 (11.9)	1	95 (1.4)	1
Male	1,739 (29.8)	0.99 (0.76–1.28)	1,985 (38.8)	**1.39 (1.06–1.81)**	889 (18.4)	**1.97 (1.46–2.66)**	160 (3.1)	**2.82 (1.56–5.09)**
Ethnicity/skin color								
White	2,763 (35.0)	1	2,709 (37.1)	1	1,009 (14.2)	1	122 (1.5)	1
Mixed race	1,278 (33.1)	1.24 (0.91–1.68)	1,278 (36.5)	1.28 (0.95–1.73)	496 (18.3)	**1.68 (1.16–2.44)**	62 (2.8)	**2.41 (1.17–4.97)**
Black	254 (36.5)	0.94 (0.51–1.74)	226 (33.7)	0.82 (0.43–1.55)	84 (11.9)	0.76 (0.39–1.48)	17 (4.3)	2.59 (0.92–7.26)
Other	185 (37.9)	0.98 (0.49–1.93)	153 (33.6)	0.81 (0.37–1.77)	71 (11.0)	0.70 (0.33–1.49)	9 (3.8)	2.27 (0.45–11.45)
Marital status								
Spouse	2,749 (35.9)	1	2,731 (37.9)	1	1,072 (13.9)	1	152 (2.1)	1
No spouse	2,558 (32.9)	**0.70 (0.55–0.89)**	2,474 (36.1)	**0.73 (0.57–0.93)**	926 (15.8)	0.87 (0.65–1.16)	99 (2.0)	0.73 (0.40–1.36)
Education level (years)								
≥ 12	1,777 (36.8)	1	1,578 (34.6)	1	588 (12.4)	1	91 (1.9)	1
9 to 11	1,596 (35.6)	0.79 (0.60–1.03)	1,461 (34.3)	**0.66 (0.51–0.87)**	569 (13.6)	**0.65 (0.47–0.88)**	85 (3.1)	1.25 (0.65–2.38)
0 to 8	2,059 (33.7)	0.77 (0.58–1.01)	2,279 (38.6)	**0.63 (0.48–0.83)**	889 (15.8)	**0.55 (0.40–0.76)**	79 (1.9)	0.73 (0.39–1.36)
Work in the last three months								
Yes	1,479 (35.0)	1	1,413 (37.1)	1	448 (12.3)	1	71 (1.5)	1
No	3,953 (34.6)	**1.36 (1.03–1.78)**	3,905 (36.9)	**1.36 (1.03–1.80)**	1,598 (15.7)	**1.76 (1.26–2.44)**	184 (2.4)	**2.16 (1.09–4.27)**
Private health insurance plan								
Yes	3,583 (35.7)	1	3,353 (35.1)	1	1,318 (14.2)	1	150 (1.9)	1
No	1,830 (336)	**1.13 (1.00–1.28)**	1,942 (39.1)	**1.23 (1.09–1.39)**	718 (15.2)	**1.20 (1.04–1.39)**	104 (2.4)	1.30 (0.98–1.74)
Self-assessment of health								
Very good/good	3,343 (36.5)	1	2,833 (34.5)	1	1,017 (13.6)	1	127 (1.9)	1
Average	1,750 (33.8)	1.30 (0.99–1.70)	1,969 (38.5)	1.56 (1.19–2.05)	807 (15.5)	**1.60 (1.16–2.20)**	100 (2.5)	**1.90 (1.02–3.56)**
Poor/very poor	222 (24.0)	**1.98 (1.08–3.66)**	380 (48.6)	4.26 (2.32–7.83)	158 (20.6)	**4.59 (2.37–8.89)**	837 (2.3)	**3.67 (1.35–9.93)**

In bold: statistically significant associations.

In adults, there were greater chances of co-occurrence in men, mainly of three or more factors (OR = 5.38, 95%CI 4.32–6.71), in those who did not have health insurance, and in those who assessed their health as very poor. In the older participants, the male sex was also positively associated with co-occurrence, but to a lesser extent. The chances of co-occurrence of two and three factors or more were higher in those who reported not performing any occupational activity in the past three months, in those who had no health insurance, and in those who assessed their health as average and poor or very poor ( Table 3 ).

**Table 3:** Multinomial logistic regression for multiple occurrence and co-occurrence of risk factors (relative to the absence of factors) in adults and older people. Vigitel, Brazil, 2015.

Variable	Adults			Older people		
	
	One	Two	Three or more	One	Two	Three or more
					
	OR (95%CI)	OR (95%CI)	OR (95%CI)	OR (95%CI)	OR (95%CI)	OR (95%CI)
Sex						
Female	1	1	1	1	1	1
Male	**1.39 (1.14–1.70)**	**2.28 (1.87–2.78)**	**5.38 (4.32–6.71)**	0.85 (0.64–1.14)	**1.36 (1.01–1.83)**	**2.58 (1.86–3.59)**
Marital status						
Spouse				1	1	1
No spouse				**0.65 (0.50–0.85)**	0.78 (0.59–1.04)	1.12 (0.82–1.54)
Work in the last three months						
Yes				1	1	1
No				1.27 (0.96–1.67)	**1.39 (1.04–1.86)**	**2.07 (1.47–2.92)**
Private health insurance plan						
Yes	1	1	1	1	1	1
No	1.04 (0.96–1.14)	1.14 (1.05–1.24)	1.10 (1.00–1.22)	1.12 (0.98–1.28)	**1.17 (1.03–1.34)**	1.13 (0.97–1.32)
Self-assessment of health						
Very good/good	1	1	1	1	1	1
Average	1.62 (1.32–1.99)	**2.03 (1.66–2.49)**	**3.12 (2.49–3.92)**	1.23 (0.92–1.64)	**1.50 (1.12–2.01)**	**1.59 (1.14–2.20)**
Poor/very poor	2.33 (1.34–4.06)	**4.82 (2.78–8.36)**	**8.26 (4.49–15.18)**	1.76 (0.95–3.28)	**3.86 (2.07–7.21)**	**4.29 (2.16–8.50)**

OR: Odds ratio adjusted by multiple multinomial logistic regression, considering any risk factor as the reference category (30,767 adults and 14,342 older individuals were included in the final models).

In bold: statistically significant associations.

Table 4 shows the prevalence of co-occurrence of two factors (ten combinations from the five factors evaluated) and the odds ratio for the association between them. A higher chance of alcohol abuse and poor diet was observed among smokers for both age groups. Among the adults, alcohol consumption was higher in those who were overweight (OR = 1.17, 95%CI 1.03–1.32), and poor diet, in those who were physically inactive (OR = 1.57; 95%CI 1.32–1.87).

**Table 4:** Prevalence and odds ratio (crude and adjusted) for the co-occurrence of two risk factors in adults and older people. Vigitel, Brazil, 2015.

Combination of risk factors*	Adults					Older people				
	
	%	OR_crude_	95%CI	OR_adjusted_	95%CI	%	OR_crude_	95%CI	OR_adjusted_	95%CI
Smoking and overweight	10.3	0.97	0.82–1.14	**0.83**	**0.70–0.98**	6.7	**0.51**	**0.39–0.69**	**0.51**	**0.38–0.69**
Smoking and physical inactivity	11.5	1.10	0.88–1.39	1.06	0.84–1.35	8.5	0.92	0.71–1.19	0.94	0.72–1.23
Smoking and use of alcohol	22.5	**3.44**	**2.91–4.07**	**3.52**	**2.93–4.24**	21.8	**3.14**	**2.14–4.61**	**2.94**	**1.95–4.44**
Smoking and poor diet	11.1	**1.53**	**1.23–1.90**	**1.50**	**1.20–1.87**	10.5	**1.61**	**1.19–2.16**	**1.51**	**1.13–2.01**
Smoking and physical inactivity	52.3	0.98	0.85–1.12	0.97	0.85–1.12	62.6	1.12	0.94–1.32	1.11	0.94–1.31
Overweight and use of alcohol	57.0	**1.24**	**1.10–1.39**	**1.17**	**1.03–1.32**	64.4	1.18	0.84–1.66	1.19	0.84–1.70
Poor diet and overweight	52.4	0.98	0.88–1.08	0.95	0.85–1.06	61.6	1.11	0.95–1.30	1.12	0.97–1.31
Physical inactivity and use of alcohol	10.6	**0.78**	**0.65–0.92**	**0.75**	**0.63–0.90**	28.9	0.86	0.60–1.23	0.93	0.65–1.33
Physical inactivity and poor diet	13.1	**1.57**	**1.32–1.87**	**1.57**	**1.32–1.87**	32.1	1.11	0.95–1.30	1.10	0.94–1.28
Poor diet and use of alcohol	21.2	**1.70**	**1.46–1.98**	**1.45**	**1.24–1.70**	7.7	**2.15**	**1.51–3.07**	**1.96**	**1.36–2.82**

*OR estimated in relation to the first risk factor. Odds ratio adjusted for sex, education and access to private health insurance.

In bold: statistically significant associations.

## DISCUSSION

This study verified the co-occurrence of the major risk factors among adults and the older population in the Brazilian capitals and the Federal District. High rates of co-occurrence were observed, corresponding to 57.3% in adults and 53.8% in older people, especially among men, those who did not have health insurance and those who assessed their health as very poor. In a study conducted in Florianópolis with adults aged from 20 to 59 years old considering smoking, alcohol abuse, poor diet and physical inactivity, the occurrence of two or more factors corresponded to 59.8%^16^ . Regarding the older population (≥ 60 years old), a study conducted in the urban area of Pelotas considering smoking, alcohol consumption, overweight and physical inactivity found that 50.9% reported two or more factors^13^ .

In the United States, the prevalence found by Liu et al.^22^ corresponded to 24.3% and 35.4% for two and three risk factors, respectively, in the adult population (≥ 21 years old). It should be noted that, in addition to smoking, physical activity, alcohol consumption and BMI, sleep time was also considered. Several national^13 , 16^ and international^15 , 22 , 23^ studies have identified the accumulation of risk factors in population subgroups. In a study that included smoking, alcohol abuse, low consumption of fruits and vegetables and physical inactivity in the population aged from 16 to 64 years old, the prevalence of two risk factors was 39.9% in men and 43.3% in women^15^ . In the study by Stenholm et al.^23^ with data from four prospective cohorts (England, Finland, France and Sweden) evaluating smoking, physical inactivity and obesity as predictors of healthy life expectancy and expected years of life free of chronic condition in those aged from 50 to 75 years old, it was found that the occurrence of two factors ranged from 9.97% (English Longitudinal Study of Ageing – ELSA) to 13.64% (Finnish Public Sector Study) in men, and from 7.76% (Swedish Longitudinal Occupational Survey of Health – SLOSH) to 13.06% (ELSA) in women. A health survey conducted in Sweden (Stockholm County Council’s) with population aged from 30 to 65 years old including smoking, alcohol abuse, low levels of physical activity and unhealthy diet found a prevalence of 31.1%, 11.2% and 1.8% for two, three and four risk factors, respectively^24^ . However, the risk factors considered and methodological differences between the studies do not allow a direct comparison with the findings of this study.

Among the older population, the occurrence of three risk factors in the North region was higher than in the Southeast. On the other hand, lower occurrence of four or more factors was found in the Northeast. The Brazilian population has been aging unevenly. Residents of the North and Northeast regions live less than the national average, and older people in particular have lower healthy life expectancy^25^ . Despite the lack of resources for health and other social policies, these areas should be prioritized when addressing the potential determinants of health inequalities that influence the reduction in mortality and add years to the life expectancy at birth^26^ .

A systematic review confirms that men accumulate more risk behaviors than women^27^ , as do recent national studies^13 , 16 , 28^ . Male behavior is determinant in the health-disease process of men^29^ . Women still tend to adhere more to health promotion and prevention practices, and seek health services more often^28^ .

In Brazil, only a quarter of the population has the necessary income or a job that allows them access to private health insurance^17^ . Vigitel’s data demonstrate that individuals with health insurance smoke less, practice more physical activity and eat more fruits and vegetables. An association between access to private health insurance and higher education level and income may be noted, not being necessarily related to access to health services. The Brazilian Unified Health System universalizes the offering of services with equity^30^ , but there are still challenges to ensure full universality without barriers.

This study noted greater co-occurrence of risk factors in the older participants who did not work. The maintenance of paid work in this subgroup represents the continuity of the individual’s complex executive function and is a mechanism of social support. The increase in income positively affects active aging, providing financial autonomy in relation to health, social and dietary needs^31^ . There is the possibility of reverse causality, since greater co-occurrence of risk factors may be associated with diseases and disabilities that prevent older adults from working.

The self-assessment of health integrates the individual’s biological, psychological and social perception. It is an indicator of quality of life, morbidity, functional decline and mortality^32^ . Two or more risk factors were associated with worse health assessment. In the Brazilian adult population (≥ 20 years), it was observed that the likelihood of individuals perceiving their health as poor or very poor was 5.27 times higher for those who reported one or more chronic diseases^25^ . Health-related behaviors are determinants of NCD and relate to the subjective assessment of health. Thus, a better understanding of the mechanisms involved in the impact of risk behaviors on health helps widen the approach to the promotion of protective behaviors.

In the analysis of the combination of simultaneous factors, some combinations were more prevalent, such as overweight associated with physical inactivity, alcohol use and improper diet (over 50%). The revision of the National Health Promotion Policy represents the Brazilian government’s efforts to ensure the intersectoral nature of public policies and comprehensiveness of health care, emphasizing healthy eating, physical activity and reduction in alcohol abuse as priority for the actions of promotion of health and healthy lifestyles^33^ . Numerous evidence indicates the importance of implementing regulatory measures for the control of risk factors associated with NDC^34^ . These measures implemented by the State are cost-effective, acting on the environment and regulating marketing practices, the availability and supply of services, the taxation of products deemed as harmful to health and food labeling^34 , 35^ .

The high prevalence of overweight in the population demands immediate action on the part of health services. Although there have been numerous advances in the food industry in the country, such as the development of the *Guia Alimentar para a População Brasileira* (Food Guide for the Brazilian Population, 2014), the encouragement of breastfeeding and the *Plano de Ação de Enfrentamento das DCNT* (Plan of Action Against NCD), preventing the growth of obesity is still a major challenge^37^ . WHO recommends measures such as taxing ultra-processed food, offering subsidies for healthy foods and prohibiting food marketing directed at children^34 , 35^ . Mexico adopted a law that taxes ultra-processed foods and beverages with high sugar content in 2013, reducing the consumption of soft drinks by 10% and increasing the consumption of water by 15%^34^ . In Australia, a study of cost-effectiveness of interventions to reduce salt intake, for example, identified that while educational programs are effective, mandatory government measures setting limits to the use of salt by industries can be up to twenty times more effective^36^ .

Still in relation to the reduction of risk factors associated with NCD, Brazil is considered a global example of reduction in the prevalence of smoking^38 , 39^ , having implemented measures such as the prohibition of tobacco advertising, the adoption of the *Convenção-Quadro para o Controle do Tabaco* (Framework Convention on Tobacco Control) in 2006, the approval of Law no. 12,546 of 2011 and presidential decree 2014 establishing smoke-free environments, the increase in the taxation of cigarettes, among others^39^ .

In this study, compared with nonsmokers, smokers had a lower prevalence of overweight. Studies show the inverse relationship between cigarette smoking and body weight attributed to the action of nicotine, caused both by the increase in adrenergic activity and energy expenditure, contributing to the reduction in body weight, and by the release of dopamine and serotonin, which act on the control and regulation of appetite in the hypothalamus^40^ . Smokers were more likely to abuse alcohol compared to non-smokers in both age groups. A study carried out in Portugal with adults (≥ 19 years old) showed higher consumption of all alcoholic beverages evaluated (beer, wine, whiskey and brandy) among smokers. In addition, the adults and older people who reported alcohol abuse were at a higher chance of having an improper diet, with less intake of vegetables and fruits in both sexes^41^ . Data from the National Health and Nutrition Examination Survey show a significant association between alcohol consumption and worse quality of diet in adult men and women (≥ 20 years old)^42^ .

Among the limitations of this study, it should be considered that all the information was self-reported, which may result in possible information bias in relation to behaviors deemed appropriate. Regarding the sample, it was restricted to the population with a landline in their home, which may decrease the participation of the North and Northeast regions of the country due to the lower coverage rates. However, the use of weighting factors reduces the differences in populations with and without a landline^7^ .

Given the cross-sectional design of the study, in the relationships between the pairs of risk factors evaluated, OR > 1, for example, indicates that individuals who exhibit a certain behavior were more likely to be associated with the other risk factor at that moment than those who were not exposed to the first factor.

As for the impact of exposition and social behavior throughout life, Stenholm et al.^23^ point out that the presence of at least two risk factors among individuals aged from 50-75 years old reduces their healthy life expectancy in eight years, and their expected years of life free of chronic condition in six years. This study reveals demands in relation to social practices and policies in health, such as public actions for regulation of risk factors due to the high percentage of individuals with multiple risks, which at the same time identify subgroups at higher risk of NCD compared to those with only one or none of the studied factors. In this way, it emphasizes the need to adopt strategies for interventions directed toward multiple behaviors, and not focused on individual factors only.

It is also necessary to emphasize that, for the promotion of healthy lifestyles, macro-social, inter-sectoral and regulatory policies are more effective^34 , 35^ . The State’s role as regulator also of sectors other than health should be emphasized for the promotion of healthy lifestyles. In the individual health care context, it must be considered that there is a high prevalence of accumulation of risk factors in the population, and that substitute behaviors lead to the maintenance or expansion of these percentages, if the approach adopted is not extended to all these factors. Thus, comprehensiveness as a value to be sustained must be present at consultations, during the conversation in which the health care provider seeks to recognize, in addition to the explicit demands, the needs of patients^43^ . Broadening the approach used to address health issues is a fundamental and necessary condition in all health services, since the State has the duty to offer “comprehensive care, prioritizing preventive activities, without damages to health services”, as laid out in the Federal Constitution of 1988.
