# Supplementary material for: Prevalence and co-occurrence of modifiable risk factors in adults and older people
Source: Rev Saude Publica. 2019 Oct 16;53:86. doi: 10.11606/s1518-8787.2019053001142 (PMC6802949; doi:10.11606/s1518-8787.2019053001142)
Supplement: Translation

# Translation: Prevalência e coocorrência de fatores de risco modificáveis em adultos e idosos

**Authors:** Priscila Maria Stolses Bergamo Francisco, Daniela de Assumpção, Flávia Silva Arbex Borim, Caroline Senicato, Deborah Carvalho Malta

**Affiliations:** I Universidade Estadual de Campinas. Faculdade de Ciências Médicas. Departamento de Saúde Coletiva. Campinas, SP, Brasil; II Universidade Federal de Minas Gerais. Escola de Enfermagem. Belo Horizonte, MG, Brasil

**Keywords:** Adulto, Idoso, Doenças não Transmissíveis, prevenção & controle, Fatores de Risco, Comportamento Sedentário, Estilo de Vida Saudável, Conhecimentos, Atitudes e Prática em Saúde

## Abstract

**OBJETIVO:**

Estimar a coocorrência dos principais fatores de risco para doenças crônicas não transmissíveis em adultos (18 a 59 anos) e idosos (≥ 60 anos) residentes nas capitais dos estados brasileiros e Distrito Federal.

**MÉTODOS:**

Estudo transversal de base populacional com dados de 35.448 adultos e 18.726 idosos coletados no Sistema de Vigilância de Fatores de Risco e Proteção para Doenças Crônicas por Inquérito Telefônico (Vigitel) em 2015. Estimaram-se as prevalências de cada um dos cinco fatores de risco (tabagismo, excesso de peso, inatividade física, uso de álcool e alimentação não saudável), assim como sua coocorrência para as diferentes combinações possíveis, segundo variáveis socioeconômicas e autoavaliação da saúde. As associações independentes foram verificadas por meio de regressão logística multinomial para obter as estimativas do *odds ratio* (OR) e respectivos intervalos de confiança de 95%.

**RESULTADOS:**

Ao menos dois fatores de risco estavam presentes em 38,5% dos adultos e 37,0% dos idosos. Adultos e idosos do sexo masculino, que não possuíam plano de saúde privado e que avaliaram sua saúde como regular ou ruim/muito ruim apresentaram maiores chances de ter dois ou mais comportamentos de risco simultâneos. Destaca-se a maior chance de coocorrência de tabagismo e uso abusivo de álcool em adultos (OR ajustado = 3,52) e idosos (OR ajustado = 2,94).

**CONCLUSÕES:**

Foram identificados subgrupos com maior risco de desenvolver múltiplos comportamentos não saudáveis e os comportamentos mais prevalentes. Almeja-se que esses resultados contribuam para o melhor direcionamento de ações de promoção de saúde e assistência preventiva. Destaca-se que, para a adoção de hábitos de vida saudáveis, as políticas macrossociais e intersetoriais são mais efetivas.

## INTRODUÇÃO

Os fatores de risco modificáveis são ações prejudiciais que elevam a probabilidade de ocorrência da doença ou impedem a recuperação da saúde^1^ . Constituem causas componentes na determinação de doenças e condições de saúde, com impacto na incidência de morbidade e mortalidade por doenças crônicas não transmissíveis (DCNT) – principalmente as cardiovasculares, a diabetes *mellitus* e as neoplasias – em adultos e idosos^2^ .

De acordo com a Organização Mundial da Saúde (OMS), um pequeno conjunto de fatores de risco responde pela maioria das mortes por DCNT e por importante fração da carga de doenças por essas enfermidades^6^ . Tabagismo, uso abusivo de bebidas alcoólicas, inatividade física e alimentação não saudável representam os principais fatores de risco relacionados à morbimortalidade por DCNT^6 , 7^ . Os fatores de risco comportamentais provocam mudanças metabólicas, dentre as quais o excesso de peso (sobrepeso ou obesidade), responsável por 5,0% do total de óbitos causados por DCNT no mundo^6^ . Diversos estudos epidemiológicos evidenciam a contribuição desses fatores na determinação das doenças^5 , 8^ .

A presença simultânea de dois ou mais fatores de risco potencializa a ocorrência das DCNT^2 , 11^ e associa-se à mortalidade total e por causas específicas^3 , 14^ em homens e mulheres^14^ . De modo geral, os fatores de risco relacionados ao estilo de vida não ocorrem isoladamente entre os indivíduos, mas de maneira agrupada, e não são distribuídos aleatoriamente em toda a população^15^ .

Em estudo sobre o efeito de fatores de risco potencialmente modificáveis associados ao infarto do miocárdio em 52 países, o tabagismo, alimentação inadequada, hipertensão arterial, diabetes *mellitus* e estresse psicossocial responderam por 90% e 94% do risco atribuível populacional para doenças cardiovasculares entre homens e mulheres, respectivamente^11^ . No Brasil, estudo que estimou a fração de risco atribuível para 25 tipos de câncer resultante da exposição a diversos fatores de risco modificáveis (tabagismo, consumo de álcool, dieta, sobrepeso e obesidade, inatividade física, agentes ocupacionais e ambientais, entre outros) concluiu que eles serão responsáveis por 34% dos casos de câncer entre os homens e de 35% entre mulheres em 2020, e por 46% e 39% das mortes, respectivamente^10^ .

Estudos nacionais vêm considerando principalmente o tabagismo, alimentação não saudável, inatividade física no lazer, consumo de álcool e excesso de peso nas análises sobre a aglomeração de fatores de risco na população. Em adultos, a ocorrência de dois ou mais fatores foi maior nos homens e nos segmentos de menor renda *per capita* e escolaridade, além de ter diminuído com o avançar da idade, em estudo realizado por Silva et al.^16^ ; na população idosa, foi menor com o aumento da idade^13^ . Entre os fatores mencionados, o marcador de dieta inadequada geralmente utilizado é o consumo insuficiente de frutas e vegetais, apontando uma carência de informações mais abrangentes sobre o perfil alimentar.

O acesso a planos privados de saúde não tem sido incluído na avaliação da coocorrência dos fatores de risco. Esse indicador pode ser considerado para diferenciar estratos socias^17^ , visando a implementação de políticas públicas de promoção da saúde e prevenção de agravos que considerem subgrupos específicos. Também a relação entre a autoavaliação da saúde – que integra a percepção biológica, psicológica e social do indivíduo – e a coocorrência de fatores de risco ainda é pouco estudada. A prevalência simultânea dos principais fatores de risco para DCNT e sua distribuição na população adulta e idosa ainda é pouco dimensionada. A exposição a fatores de risco comportamentais se inicia precocemente^12^ , consolidando-se na vida adulta^16^ , e apresenta reflexos negativos para a saúde nas diversas fases da vida. Além dos anos potenciais de vida perdidos devido à mortalidade ou incapacidade em idades mais precoces, deve-se considerar o impacto da exposição a esses fatores ao longo da vida entre os indivíduos com idades mais avançadas. Em ambos os segmentos etários, tais condições representam importante demanda dos serviços de saúde, além do suporte familiar e de apoio social. Medidas de promoção e prevenção são mais efetivas, a depender de abordagem diferencial em relação às faixas etárias e demais características sociodemográficas.

Partindo da hipótese de que existe uma elevada prevalência de aglomeração de fatores de risco na população e de que sua ocorrência varia de acordo com as características sociodemográficas e percepção de saúde, o objetivo do estudo foi estimar a prevalência da coocorrência dos principais fatores de risco para DCNT em adultos e idosos residentes nas capitais dos estados brasileiros e no Distrito Federal em 2015, bem como verificar sua associação com características sociodemográficas e autoavaliação da saúde.

## MÉTODOS

Estudo transversal de base populacional com adultos (18 a 59 anos) e idosos (≥ 60 anos) residentes nas capitais dos estados brasileiros e no Distrito Federal (DF). Os dados foram obtidos a partir de amostra probabilística de moradores em domicílios servidos por pelo menos uma linha de telefone fixo (n = 54.174) e coletados pelo Sistema de Vigilância de Fatores de Risco e Proteção para Doenças Crônicas por Inquérito Telefônico (Vigitel) em 2015.

O Vigitel define um tamanho mínimo de aproximadamente 2.000 indivíduos na amostra de cada cidade para estimar com coeficiente de confiança de 95% e erro máximo de 2% a frequência dos principais fatores de risco para doenças crônicas não trasmissíveis^18^ . Na primeira etapa de amostragem, realizou-se um sorteio sistemático e estratificado por código de endereçamento postal, a partir de cadastros telefônicos de empresas que servem as 26 capitais e o DF^7^ . Após identificação das linhas elegíveis para o sistema, sorteou-se um dos adultos (≥ 18 anos) residentes no domicílio selecionado. Em 2015, o Vigitel identificou 76.703 linhas elegíveis, obtendo 54.174 entrevistas^7^ .

Os pesos atribuídos aos indivíduos selecionados inicialmente consideram dois fatores: o inverso do número de linhas telefônicas no domicílio, que corrige a maior chance que indivíduos de domicílios com mais de uma linha tiveram de ser selecionados, e o número de adultos no domicílio do entrevistado, o qual corrige a menor chance que indivíduos de domicílios onde residem mais pessoas tiveram de ser sorteados. Pelo produto desses fatores, obtém-se um peso amostral que possibilita calcular estimativas confiáveis para a população adulta com telefone em cada cidade^7^ .

Finalmente, aplicou-se o peso de pós-estratificação^19^ , calculado com base em 36 categorias de análise por sexo, faixa etária e nível de instrução, permitindo igualar a composição sociodemográfica estimada para a população adulta com telefone em cada capital à composição sociodemográfica que se estima para a população adulta total^7^ .

### Fatores de Risco

Os fatores de risco analisados foram selecionados por sua importância para a determinação da carga total de doença estimada pela OMS para a região das Américas^20^ . Além deles, elaborou-se um indicador de alimentação não saudável considerando um conjunto de alimentos relacionados à proteção e risco para DCNT.

### Tabagismo

Expresso pelo percentual de fumantes entre os indivíduos entrevistados. Considerou-se fumante os que responderam positivamente à questão “O(A) sr.(a) fuma?”, independentemente do número de cigarros e da duração do tabagismo.

### Excesso de Peso ou Obesidade

Percentual de indivíduos com índice de massa corporal (IMC) ≥ 25 kg/m^2, 21^ , calculado pelo peso (kg) dividido pelo quadrado da altura em metros, ambos autorreferidos. Quando os entrevistados desconheciam o seu peso ou sua altura, os valores dessas medidas foram imputados pelo uso da técnica *hot deck* ^7^ .

### Inatividade Física

Percentual de indivíduos que não praticaram qualquer atividade física no tempo livre nos últimos três meses e que não realizaram esforços físicos intensos no trabalho, não se deslocaram para o local de trabalho ou estudo caminhando ou de bicicleta por pelo menos 20 minutos no percurso de ida e volta e não realizaram limpeza pesada de sua casa^7^ .

### Consumo Abusivo de Bebidas Alcoólicas

Percentual de indivíduos que consumiram bebidas alcoólicas abusivamente (cinco ou mais doses para homens e quatro ou mais doses para mulheres) pelo menos uma vez nos últimos 30 dias. No Vigitel, uma dose de bebida corresponde a uma lata de cerveja, uma taça de vinho ou uma dose de cachaça, *whisky* ou qualquer outra bebida alcoólica destilada.

### Alimentação Não Saudável

O indicador de alimentação não saudável foi elaborado a partir de um conjunto de alimentos considerados de proteção para doenças crônicas (frutas, hortaliças cruas e cozidas, leite e feijão) ou de risco (doces, carnes vermelhas, refrigerantes e outras bebidas adoçadas). Dependendo do alimento e da frequência de consumo, foram atribuídos entre zero e quatro pontos. Calculou-se o escore de forma inversa, ou seja, receberam pontuação mínima (zero) os alimentos considerados de proteção ingeridos diariamente e os de risco consumidos quase nunca ou nunca. A pontuação máxima (quatro pontos) foi conferida aos alimentos de proteção quase nunca ou nunca consumidos e aos alimentos de risco ingeridos diariamente ( Quadro 1 ). O escore total compreendeu a soma dos itens alimentares, variando de 0 (melhor) a 32 pontos (pior qualidade da alimentação). A pontuação total foi categorizada em tercis da distribuição, e em seguida foram agrupados os indivíduos pertencentes ao 2º e 3º tercil (≥ 13 pontos) para compor uma variável dicotômica para alimentação não saudável (sim ou não).

**Quadro 1:** Escore para o consumo de alimentos de forma não saudável. Vigitel, Brasil, 2015.

Alimentos	0	1	2	3	4
Feijão	Todos os dias	5 a 6 dias por semana	3 a 4 dias por semana	1 a 2 dias por semana	Nunca ou quase nunca
Frutas	Todos os dias	5 a 6 dias por semana	3 a 4 dias por semana	1 a 2 dias por semana	Nunca ou quase nunca
Hortaliças cruas^a^	Todos os dias	5 a 6 dias por semana	3 a 4 dias por semana	1 a 2 dias por semana	Nunca ou quase nunca
Hortaliças cozidas^b^	Todos os dias	5 a 6 dias por semana	3 a 4 dias por semana	1 a 2 dias por semana	Nunca ou quase nunca
Leite	Todos os dias	5 a 6 dias por semana	3 a 4 dias por semana	1 a 2 dias por semana	Nunca ou quase nunca
Carne vermelha^c^	Nunca ou quase nunca	1 a 2 dias por semana	3 a 4 dias por semana	5 a 6 dias por semana	Todos os dias
Refrigerante ou suco artificial	Nunca ou quase nunca	1 a 2 dias por semana	3 a 4 dias por semana	5 a 6 dias por semana	Todos os dias
Doces^d^	Nunca ou quase nunca	1 a 2 dias por semana	3 a 4 dias por semana	5 a 6 dias por semana	Todos os dias

^a^Salada de alface e tomate ou qualquer outra salada de hortaliça crua.

^b^Consumo de hortaliças cozidas com a comida ou na sopa, como couve, cenoura, chuchu, berinjela e abobrinha, excluindo batata, mandioca ou inhame.

^c^De boi, porco ou cabrito.

^d^Sorvetes, chocolates, bolos, biscoitos e outros.

### Análises Estatísticas

As variáveis sociodemográficas consideradas foram: macrorregião geográfica do país (Norte, Nordeste, Centro-Oeste, Sul e Sudeste), sexo (masculino e feminino), cor da pele/raça (branca, parda, preta e outras), situação conjugal (com cônjuge e sem cônjuge), escolaridade (0–8, 9–11 e ≥12 anos de estudo), realização de atividade ocupacional nos últimos três meses (sim e não), plano de saúde (sim e não). Também foi considerada a autoavaliação da saúde (muito boa ou boa, regular e ruim ou muito ruim). Os fatores de risco foram codificados como variáveis binárias (presença = 1 e ausência = 0). Pela soma dos comportamentos individuais, gerou-se um escore variando de 0 a 5, baseado na distribuição observada. A variável criada, número de fatores de risco, foi categorizada em nenhum, um, dois, três e quatro ou mais.

Inicialmente estimaram-se as prevalências do número de fatores, segundo variáveis sociodemográficas e autoavaliação da saúde para ambos os grupos. As associações foram verificadas pelas razões de chances ( *odds ratio* ; OR) brutas e os respectivos intervalos de confiança de 95% (IC95%) – regressão logística multinomial; categoria de referência: ausência de fatores de risco.

Em seguida, realizou-se análise múltipla selecionando como variável dependente (politômica) o número de fatores (nenhum, um, dois, três ou mais), obtendo-se as estimativas ajustadas do OR e respectivos IC95%. Foram incluídas no modelo inicial todas as variáveis com associação na análise simples (p < 0,20), e permaneceram no modelo final aquelas com p < 0,05. Nesta análise, os grupos foram simultaneamente comparados com a categoria de referência (nenhum dos cinco fatores de risco).

Adicionalmente, também foram estimadas as prevalências da coocorrência de dois fatores (10 combinações possíveis, a partir dos cinco fatores avaliados). As razões de chances (OR) foram obtidas por regressão logística binária, expressa pela equação:

$$OR\;=\;\frac{{𝑓}_{11}\;𝒙{𝑓}_{00}}{{𝑓}_{10}\;𝒙\;{𝑓}_{01}\;}$$

onde *f*_11_corresponde ao número de indivíduos que referiu ambos os fatores, *f*_00_os respondentes que não apresentaram nenhum, *f*_10_aqueles em que o primeiro fator esteve presente e *f*_01_os que relataram apenas a presença do outro fator. Assim, foi criada uma variável dicotômica, cuja categoria de interesse foi a presença de ambos os fatores de risco (sim ou não). As análises foram realizadas no Stata 14.0, considerando-se o delineamento amostral complexo.

Os objetivos do inquérito foram apresentados aos indivíduos no contato telefônico, e o formulário de consentimento livre e esclarecido foi substituído pelo consentimento verbal. O estudo foi aprovado pela Comissão Nacional de Ética em Pesquisa em Seres Humanos do Ministério da Saúde, sob o parecer nº 355.590 de 26 de junho de 2013.

## RESULTADOS

A média de idade foi de 40,0 anos (DP = 12,1) nos adultos e de 70,3 anos (DP = 7,9) nos idosos, com maior percentual de mulheres (52,6% e 60,7%, respectivamente). Na população adulta, 51,0% se declararam pardos ou pretos e, entre os idosos, 33,8%. A proporção de indivíduos sem cônjuge foi maior entre os adultos (53,1%) que entre os idosos (42,9%). Observou-se menor escolaridade entre os idosos para todas as categorias avaliadas; enquanto cerca de 30,0% dos adultos referiram escolaridade ≥ 12 anos, nos idosos esse percentual foi de apenas 17,0%. Quanto à avaliação subjetiva da saúde, 67,3% dos adultos e 53,8% dos idosos consideravam sua saúde como muito boa ou boa, 28,4% e 38,8% como regular, e 4,3% e 7,4% como ruim ou muito ruim, respectivamente. Exceto para o plano de saúde, nas demais variáveis observaram-se diferenças estatisticamente significativas entre adultos e idosos (p < 0,05). Também foram constatadas diferenças na avaliação dos fatores de risco individuais. Os adultos apresentaram maiores prevalências de tabagismo, uso abusivo de álcool e alimentação não saudável (p < 0,001) (dados não apresentados).

Verificou-se elevado percentual de indivíduos com ao menos dois fatores de risco (38,5%). Observaram-se dois comportamentos de risco em 40,0% dos homens, e elevadas chances de ocorrência de três (OR = 4,54; IC95% 3,63–5,67) e quatro ou mais fatores (OR = 5,93; IC95% 4,16–8,45). A coocorrência de dois ou mais fatores foi mais elevada naqueles com menor escolaridade e nos que não possuíam plano de saúde à época da pesquisa. Menor prevalência de três e quatro ou mais fatores foi constatada entre os adultos que não estavam trabalhando. Em relação à avaliação subjetiva da saúde, maior prevalência do acúmulo de fatores de risco foi verificada entre os que consideraram sua saúde como regular e ruim ou muito ruim ( Tabela 1 ).

**Tabela 1:** Prevalência e análise univariada da associação entre o número de fatores de risco para DCNT, variáveis sociodemográficas e autoavaliação de saúde na população adulta. Vigitel, Brasil, 2015.

Variável	Um		Dois		Três		Quatro ou mais	
			
	n (%)	OR (IC95%)	n (%)	OR (IC95%)	n (%)	OR (IC95%)	n (%)	OR (IC95%)
Total	11.062 (34,4)		11.608 (38,5)		4.362 (15,5)		849 (3,3)	
Região geográfica								
Sudeste	1.495 (34,0)	1	1.554 (38,4)	1	584 (16,0)	1	134 (3,5)	1
Nordeste	3.711 (35,1)	0,97 (0,80–1,18)	3.847 (39,0)	0,95 (0,78–1,16)	1.417 (14,7)	0,86 (0,69–1,09)	259 (2,7)	0,73 (0,51–1,03)
Norte	3.284 (34,4)	1,16 (0,93–1,44)	3.438 (40,3)	1,20 (0,97–1,50)	1.259 (15,2)	1,09 (0,85–1,41)	234 (3,1)	0,99 (0,62–1,59)
Centro-Oeste	1.468 (34,5)	0,81 (0,61–1,08)	1.537 (36,6)	0,76 (0,57–1,02)	629 (15,3)	0,77 (0,56–1,06)	131 (3,6)	0,80 (0,50–1,30)
Sul	1.104 (34,1)	0,93 (0,73–1,17)	1.232 (38,4)	0,93 (0,73–1,17)	473 (15,8)	0,92 (0,70–1,20)	91 (3,2)	0,83 (1,55–1,25)
Sexo								
Feminino	7.423 (40,7)	1	6.387 (37,2)	1	1.582 (9,5)	1	245 (1,7)	1
Masculino	3.639 (27,7)	**1,34 (1,10–1,63)**	5.221 (40,0)	**2,12 (1,74–2,58)**	2.780 (21,8)	**4,54 (3,63–5,67)**	604 (5,0)	**5,93 (4,16–8,45)**
Cor da pele/raça								
Branca	4.313 (34,7)	1	4.469 (38,4)	1	1.701 (14,6)	1	387 (3,7)	1
Parda	4.549 (34,7)	1,11 (0,92–1,33)	4.736 (40,1)	1,16 (0,96–1,39)	1.746 (14,9)	1,13 (0,91–1,41)	300 (2,6)	0,76 (0,54–1,08)
Preta	773 (35,4)	1,13 (0,78–1,64)	811 (37,2)	1,07 (0,74–1,56)	318 (16,0)	1,22 (0,80–1,85)	59 (3,6)	1,07 (0,58–2,00)
Outras	490 (32,8)	0,70 (0,46–1,06)	514 (33,5)	**0,65 (0,43–0,98)**	174 (19,0)	0,97 (0,59–1,57)	26 (3,1)	0,62 (0,25–1,58)
Estado civil								
Com cônjuge	5.286 (32,8)	1	6.101 (40,2)	1	2.263 (15,6)	1	399 (3,3)	1
Sem cônjuge	5.562 (35,7)	1,05 (0,89–1,25)	5.330 (37,2)	0,89 (0,75–1,06)	2.043 (15,5)	0,96 (0,79–1,18)	434 (3,1)	0,91 (0,66–1,24)
Escolaridade (em anos)								
≥ 12	4.953 (36,4)	1	4.915 (37,9)	1	1.838 (14,1)	1	349 (2,6)	1
9 a 11	4.524 (35,5)	1,04 (0,88–1,23)	4.724 (37,6)	1,06 (0,89–1,25)	1.779 (15,3)	1,15 (0,95–1,40)	332 (3,2)	1,30 (0,94–1,81)
0 a 8	1.585 (30,1)	1,00 (0,77–1,30)	1.969 (40,9)	**1,31 (1,01–1,70)**	745 (17,5)	**1,50 (1,11–2,03)**	168 (4,1)	**1,90 (1,25–2,89)**
Trabalho nos últimos três meses								
Sim	7.658 (33,6)	1	8.246 (38,7)	1	3.265 (16,4)	1	622 (3,4)	1
Não	3.404 (36,5)	0,93 (0,77–1,12)	3.362 (38,2)	0,84 (0,70–1,01)	1.097 (13,2)	**0,69 (0,55–0,86)**	227 (2,8)	**0,70 (0,49–0,99)**
Plano privado de saúde								
Sim	6.283 (36,3)	1	6.357 (36,5)	1	2.434 (14,9)	1	461 (2,9)	1
Não	4.764 (32,4)	1,08 (0,99–1,18)	5.224 (40,6)	**1,21 (1,11–1,31)**	1.920 (16,1)	**1,19 (1,08–1,32)**	388 (3,7)	**1,28 (1,10–1,50)**
Autoavaliação da saúde								
Muito boa/boa	8.052 (36,4)	1	7.651 (37,7)	1	2.693 (13,2)	1	492 (2,9)	1
Regular	2.651 (31,2)	**1,59 (1,30–1,95)**	3.380 (39,7)	**1,95 (1,60–2,39)**	1.424 (20,3)	**2,86 (2,27–3,60)**	295 (3,5)	**2,20 (1,59–3,06)**
Ruim/muito ruim	290 (23,4)	**2,22 (1,28–3,88)**	507 (46,2)	**4,24 (2,45–7,35)**	214 (20,2)	**5,30 (2,69–10,43)**	52 (7,4)	**8,72 (3,93–19,35)**

Em negrito: associações estatisticamente significativas.

Nos idosos, a coocorrência de três fatores de risco foi maior entre os residentes na região Norte e, para quatro ou mais fatores, foi menor no Nordeste, em relação à região Sudeste do país. Os diferenciais por sexo se mantiveram, com maiores prevalências entre os homens. A coocorrência de três ou mais fatores de risco foi maior nos pardos, comparados aos brancos. Também foi mais elevada naqueles que não exerciam uma atividade ocupacional, que não tinham plano de saúde e que avaliaram sua saúde como regular e ruim ou muito ruim. Menor prevalência de dois fatores foi observada nos que viviam sem cônjuge (OR = 0,73; IC95% 0,57–0,93) e, naqueles com até 11 anos de estudo, a coocorrência de dois ou três fatores foi significativamente menor ( Tabela 2 ).

**Tabela 2:** Prevalência e análise univariada da associação entre o número de fatores de risco para DCNT, variáveis sociodemográficas e autoavaliação de saúde na população idosa. Vigitel, Brasil, 2015.

Variáveis	Um		Dois		Três		Quatro ou mais	
			
	n (%)	OR (IC95%)	n (%)	OR (IC95%)	n (%)	OR (IC95%)	n (%)	OR (IC95%)
Total	5.432 (34,7)		5.318 (37,0)		2.046 (14,7)		255 (2,1)	
Região geográfica								
Sudeste	997 (34,9)	1	931 (37,0)	1	336 (14,2)	1	59 (2,5)	1
Nordeste	1.546 (34,6)	0,99 (0,77–1,27)	1.533 (36,6)	0,98 (0,76–1,27)	622 (16,0)	1,12 (0,83–1,52)	62 (1,3)	**0,54 (0,30–0,97)**
Norte	1.051 (33,3)	1,26 (0,90–1,78)	1.047 (38,3)	1,37 (0,97–1,93)	391 (16,8)	**1,57 (1,06–2,32)**	57 (2,8)	1,49 (0,71–3,13)
Centro-Oeste	1.060 (35,0)	0,85 (0,64–1,14)	1.001 (35,2)	0,81 (0,60–1,08)	397 (14,4)	0,86 (0,61–1,21)	44 (1,9)	0,63 (0,33–1,23)
Sul	778 (34,8)	1,03 (0,77–1,37)	806 (38,7)	1,08 (0,81–1,44)	300 (13,8)	1,00 (0,71–1,41)	33 (1,6)	0,67 (0,35–1,29)
Sexo								
Feminino	3.693 (38,5)	1	3.333 (35,6)	1	1.157 (11,9)	1	95 (1,4)	1
Masculino	1.739 (29,8)	0,99 (0,76–1,28)	1.985 (38,8)	**1,39 (1,06–1,81)**	889 (18,4)	**1,97 (1,46–2,66)**	160 (3,1)	**2,82 (1,56–5,09)**
Cor da pele/raça								
Branca	2.763 (35,0)	1	2.709 (37,1)	1	1.009 (14,2)	1	122 (1,5)	1
Parda	1.278 (33,1)	1,24 (0,91–1,68)	1.278 (36,5)	1,28 (0,95–1,73)	496 (18,3)	**1,68 (1,16–2,44)**	62 (2,8)	**2,41 (1,17–4,97)**
Preta	254 (36,5)	0,94 (0,51–1,74)	226 (33,7)	0,82 (0,43–1,55)	84 (11,9)	0,76 (0,39–1,48)	17 (4,3)	2,59 (0,92–7,26)
Outras	185 (37,9)	0,98 (0,49–1,93)	153 (33,6)	0,81 (0,37–1,77)	71 (11,0)	0,70 (0,33–1,49)	9 (3,8)	2,27 (0,45–11,45)
Estado civil								
Com cônjuge	2.749 (35,9)	1	2.731 (37,9)	1	1.072 (13,9)	1	152 (2,1)	1
Sem cônjuge	2.558 (32,9)	**0,70 (0,55–0,89)**	2.474 (36,1)	**0,73 (0,57–0,93)**	926 (15,8)	0,87 (0,65–1,16)	99 (2,0)	0,73 (0,40–1,36)
Escolaridade (em anos)								
≥ 12	1.777 (36,8)	1	1.578 (34,6)	1	588 (12,4)	1	91 (1,9)	1
9 a 11	1.596 (35,6)	0,79 (0,60–1,03)	1.461 (34,3)	**0,66 (0,51–0,87)**	569 (13,6)	**0,65 (0,47–0,88)**	85 (3,1)	1,25 (0,65–2,38)
0 a 8	2.059 (33,7)	0,77 (0,58–1,01)	2.279 (38,6)	**0,63 (0,48–0,83)**	889 (15,8)	**0,55 (0,40–0,76)**	79 (1,9)	0,73 (0,39–1,36)
Trabalho nos últimos três meses								
Sim	1.479 (35,0)	1	1.413 (37,1)	1	448 (12,3)	1	71 (1,5)	1
Não	3.953 (34,6)	**1,36 (1,03–1,78)**	3.905 (36,9)	**1,36 (1,03–1,80)**	1.598 (15,7)	**1,76 (1,26–2,44)**	184 (2,4)	**2,16 (1,09–4,27)**
Plano privado de saúde								
Sim	3.583 (35,7)	1	3.353 (35,1)	1	1.318 (14,2)	1	150 (1,9)	1
Não	1.830 (336)	**1,13 (1,00–1,28)**	1.942 (39,1)	**1,23 (1,09–1,39)**	718 (15,2)	**1,20 (1,04–1,39)**	104 (2,4)	1,30 (0,98–1,74)
Autoavaliação da saúde								
Muito boa/boa	3.343 (36,5)	1	2.833 (34,5)	1	1.017 (13,6)	1	127 (1,9)	1
Regular	1.750 (33,8)	1,30 (0,99–1,70)	1.969 (38,5)	21,7	807 (15,5)	**1,60 (1,16–2,20)**	100 (2,5)	**1,90 (1,02–3,56)**
Ruim/muito ruim	222 (24,0)	**1,98 (1,08–3,66)**	380 (48,6)	4,26 (2,32–7,83)	158 (20,6)	**4,59 (2,37–8,89)**	837 (2,3)	**3,67 (1,35–9,93)**

Em negrito: associações estatisticamente significativas.

Nos adultos, verificaram-se maiores chances da coocorrência nos homens, principalmente de três ou mais fatores (OR = 5,38; IC95% 4,32–6,71), naqueles que não possuíam plano de saúde e nos que pior avaliaram sua saúde. Nos idosos, também o sexo masculino esteve positivamente associado à coocorrência, porém com menor magnitude. As chances de coocorrência de dois e de três fatores ou mais foram maiores nos que referiam não realizar qualquer atividade ocupacional nos últimos três meses, nos que não possuíam plano de saúde e nos que autoavaliaram a saúde como regular e ruim ou muito ruim ( Tabela 3 ).

**Tabela 3:** Regressão logística multinomial múltipla para a ocorrência e coocorrência de fatores de risco (em relação à ausência de fatores) em adultos e idosos. Vigitel, Brasil, 2015.

Variável	Adultos			Idosos		
	
	Um	Dois	Três ou mais	Um	Dois	Três ou mais
					
	OR (IC95%)	OR (IC95%)	OR (IC95%)	OR (IC95%)	OR (IC95%)	OR (IC95%)
Sexo						
Feminino	1	1	1	1	1	1
Masculino	**1,39 (1,14–1,70)**	**2,28 (1,87–2,78)**	**5,38 (4,32–6,71)**	0,85 (0,64–1,14)	**1,36 (1,01–1,83)**	**2,58 (1,86–3,59)**
Estado civil						
Com cônjuge				1	1	1
Sem cônjuge				**0,65 (0,50–0,85)**	0,78 (0,59–1,04)	1,12 (0,82–1,54)
Trabalho nos últimos três meses						
Sim				1	1	1
Não				1,27 (0,96–1,67)	**1,39 (1,04–1,86)**	**2,07 (1,47–2,92)**
Plano privado de saúde						
Sim	1	1	1	1	1	1
Não	1,04 (0,96–1,14)	1,14 (1,05–1,24)	1,10 (1,00–1,22)	1,12 (0,98–1,28)	**1,17 (1,03–1,34)**	1,13 (0,97–1,32)
Autoavaliação da saúde						
Muito boa/boa	1	1	1	1	1	1
Regular	1,62 (1,32–1,99)	**2,03 (1,66–2,49)**	**3,12 (2,49–3,92)**	1,23 (0,92–1,64)	**1,50 (1,12–2,01)**	**1,59 (1,14–2,20)**
Ruim/muito ruim	2,33 (1,34–4,06)	**4,82 (2,78–8,36)**	**8,26 (4,49–15,18)**	1,76 (0,95–3,28)	**3,86 (2,07–7,21)**	**4,29 (2,16–8,50)**

OR: *Odds ratio* ajustado por meio de regressão logística multinomial múltipla, considerando nenhum fator de risco como categoria de referência (30.767 adultos e 14.342 idosos foram incluídos nos modelos finais).

Em negrito: associações estatisticamente significativas.

Na Tabela 4 são apresentadas as prevalências da coocorrência de dois fatores (dez combinações a partir dos cinco fatores avaliados) e os *odds ratio* para a associação entre eles. Observou-se maior chance de uso abusivo de álcool e de dieta inadequada entre tabagistas para ambos os recortes etários. Entre os adultos, o uso de álcool foi maior naqueles com excesso de peso (OR = 1,17; IC95% 1,03–1,32) e a dieta inadequada, nos inativos fisicamente (OR = 1,57; IC95% 1,32–1,87).

**Tabela 4:** Prevalência e *odds ratio* (bruto e ajustado) para a coocorrência de dois fatores de risco em adultos e idosos. Vigitel, Brasil, 2015.

Combinação dos fatores de risco*	Adultos					Idosos				
	
	%	OR_bruto_	IC95%	OR_ajustado_	IC95%	%	OR_bruto_	IC95%	OR_ajustado_	IC95%
Tabagismo e excesso de peso	10,3	0,97	0,82–1,14	**0,83**	**0,70–0,98**	6,7	**0,51**	**0,39–0,69**	**0,51**	**0,38–0,69**
Tabagismo e inatividade física	11,5	1,10	0,88–1,39	1,06	0,84–1,35	8,5	0,92	0,71–1,19	0,94	0,72–1,23
Tabagismo e uso de álcool	22,5	**3,44**	**2,91–4,07**	**3,52**	**2,93–4,24**	21,8	**3,14**	**2,14–4,61**	**2,94**	**1,95–4,44**
Tabagismo e dieta inadequada	11,1	**1,53**	**1,23–1,90**	**1,50**	**1,20–1,87**	10,5	**1,61**	**1,19–2,16**	**1,51**	**1,13–2,01**
Excesso de peso e inatividade física	52,3	0,98	0,85–1,12	0,97	0,85–1,12	62,6	1,12	0,94–1,32	1,11	0,94–1,31
Excesso de peso e uso de álcool	57,0	**1,24**	**1,10–1,39**	**1,17**	**1,03–1,32**	64,4	1,18	0,84–1,66	1,19	0,84–1,70
Dieta inadequada e excesso de peso	52,4	0,98	0,88–1,08	0,95	0,85–1,06	61,6	1,11	0,95–1,30	1,12	0,97–1,31
Inatividade física e uso de álcool	10,6	**0,78**	**0,65–0,92**	**0,75**	**0,63–0,90**	28,9	0,86	0,60–1,23	0,93	0,65–1,33
Inatividade física e dieta inadequada	13,1	**1,57**	**1,32–1,87**	**1,57**	**1,32–1,87**	32,1	1,11	0,95–1,30	1,10	0,94–1,28
Dieta inadequada e uso de álcool	21,2	**1,70**	**1,46–1,98**	**1,45**	**1,24–1,70**	7,7	**2,15**	**1,51–3,07**	**1,96**	**1,36–2,82**

* OR calculado em relação ao primeiro fator de risco. *Odds ratio* ajustado por sexo, escolaridade e acesso a plano privado de saúde.

Em negrito: associações estatisticamente significativas.

## DISCUSSÃO

Neste estudo foi verificada a coocorrência dos principais fatores de risco na população adulta e idosa das capitais brasileiras e do DF. Observaram-se elevados percentuais de coocorrência, de 57,3% nos adultos e 53,8% nos idosos, especialmente entre os homens, os que não possuíam plano de saúde e os que pior avaliaram sua saúde. Em estudo realizado em Florianópolis com adultos de 20 a 59 anos considerando o tabagismo, uso abusivo de álcool, dieta inadequada e inatividade física no lazer, a ocorrência de dois ou mais fatores foi de 59,8%^16^ . Em relação aos idosos (≥ 60 anos), estudo realizado na área urbana de Pelotas considerando o tabagismo, consumo de álcool, excesso de peso e inatividade física verificou que 50,9% apresentavam dois ou mais dos fatores referidos^13^ .

Nos Estados Unidos, Liu et al.^22^ encontraram prevalências de 24,3% e de 35,4% para dois e três fatores de risco, respectivamente, na população adulta (≥ 21 anos). Note-se que, além do tabagismo, atividade física, consumo de álcool e IMC, também foi considerado o tempo de sono. Diversos estudos nacionais^13 , 16^ e internacionais^15 , 22 , 23^ têm identificado a aglomeração de fatores de risco em subgrupos populacionais. Em estudo que incluiu o tabagismo, uso abusivo de álcool, baixo consumo de frutas e hortaliças e inatividade física na população de 16 a 64 anos, a prevalência de dois fatores de risco foi de 39,9% nos homens e 43,3% nas mulheres^15^ . No estudo de Stenholm et al.^23^ com dados de quatro coortes prospectivas (Inglaterra, Finlândia, França e Suécia) avaliando o tabagismo, inatividade física e obesidade como preditores da expectativa de vida saudável e livre de doenças crônicas em indivíduos de 50 a 75 anos, foi verificado que a ocorrência de dois fatores variou de 9,97% ( *English Longitudinal Study of Ageing –* ELSA) a 13,64% ( *Finnish Public Sector Study* ) nos homens, e de 7,76% ( *Swedish Longitudinal Occupational Survey of Health* – SLOSH) a 13,06% (ELSA) nas mulheres. Inquérito de saúde realizado na Suécia ( *Stockholm County Council’s* ) com população de 30 a 65 anos incluindo tabagismo, uso abusivo de álcool, baixo nível de atividade física e dieta não saudável encontrou prevalências de 31,1%, 11,2% e 1,8% para dois, três e quatro fatores de risco, respectivamente^24^ . No entanto, os fatores de risco considerados, bem como diferenças metodológicas entre os estudos, dificultam uma comparação direta com os achados deste estudo.

Entre os idosos, a ocorrência de três fatores de risco na região Norte foi maior que no Sudeste. Por outro lado, menor ocorrência de quatro ou mais fatores foi verificada no Nordeste. O envelhecimento populacional vem ocorrendo de forma desigual no país. Os residentes das regiões Norte e Nordeste vivem menos que a média nacional e os idosos em especial apresentam menor expectativa de vida saudável^25^ . Mesmo diante da insuficiência de recursos para a saúde e para outras políticas sociais, essas regiões devem ser priorizadas no enfrentamento aos potenciais determinantes das desigualdades em saúde que influenciam na redução da mortalidade e adicionam anos à esperança de vida ao nascer^26^ .

Revisão sistemática confirma que homens acumulam mais comportamentos de risco do que mulheres^27^ , assim como recentes pesquisas nacionais^13 , 16 , 28^ . O comportamento masculino é determinante no processo saúde-doença dos homens^29^ . Mulheres ainda costumam ter mais adesão às práticas de prevenção e promoção à saúde, além de maior procura aos serviços^28^ .

No Brasil, somente um quarto da população tem poder aquisitivo ou um emprego que possibilita aderir a um plano de saúde^17^ . Dados do Vigitel demonstram que indivíduos com plano de saúde fumam menos, praticam mais atividade física e ingerem mais frutas e hortaliças. Observa-se associação entre acesso a plano de saúde privado e maior escolaridade e renda, o que não necessariamente se relaciona com o acesso aos serviços de saúde. O Sistema Único de Saúde brasileiro universaliza com equidade a oferta aos serviços^30^ , mas ainda existem desafios para garantir a completa universalidade sem barreiras.

Este estudo observou maior coocorrência de fatores de risco nos idosos que não trabalhavam. A manutenção do trabalho remunerado nesse subgrupo representa a continuidade da função executiva complexa e um mecanismo de suporte social. O incremento na renda reflete positivamente no envelhecimento ativo, proporcionando autonomia financeira em relação às necessidades de saúde, sociais e alimentares^31^ . Destaca-se a possibilidade de causalidade reversa, já que a maior coocorrência de fatores de risco pode estar associada às doenças e incapacidades que impedem o idoso de trabalhar.

A autoavaliação de saúde integra a percepção biológica, psicológica e social do indivíduo. Trata-se de um indicador de qualidade de vida, morbidade, declínio funcional e mortalidade^32^ . Dois ou mais fatores de risco associaram-se à pior avaliação da saúde. Na população adulta brasileira (≥ 20 anos), observou-se que a probabilidade de perceber a saúde como ruim ou muito ruim era 5,27 vezes maior nos que referiram uma ou mais doenças crônicas^25^ . Comportamentos relacionados à saúde são determinantes das DCNT e relacionam-se com a avaliação subjetiva da saúde. Assim, uma melhor compreensão dos mecanismos envolvidos no impacto dos comportamentos de risco sobre a saúde permite ampliar a abordagem para a promoção de comportamentos protetores.

Na análise da combinação de fatores simultâneos, alguns agrupamentos foram mais prevalentes, como o excesso de peso associado à inatividade física, ao uso de álcool e à dieta inadequada (acima de 50%). A revisão da Política Nacional de Promoção da Saúde representa o esforço do governo brasileiro para garantir a intersetorialidade das políticas públicas e a integralidade da atenção à saúde, destacando a alimentação saudável, a prática de atividades físicas e a redução do uso abusivo de álcool como prioritários para as ações de promoção da saúde e dos modos de vida^33^ . Inúmeras evidências apontam a importância de implantar medidas regulatórias para o controle dos fatores de risco de DCNT^34^ . Essas medidas instituídas pelo Estado, intervindo sobre o ambiente, regulando práticas de comercialização, disponibilidade e oferta de serviços, taxação de produtos nocivos à saúde e rotulagem de alimentos, são custo-efetivas^34 , 35^ .

Destaca-se que a elevada prevalência de excesso de peso na população brasileira demanda medidas imediatas nos serviços de saúde. Embora tenham ocorrido inúmeros avanços na área da alimentação no país, como a elaboração do Guia Alimentar para a População Brasileira (2014), o incentivo ao aleitamento materno e o Plano de Ação de Enfrentamento das DCNT, ainda são grandes os desafios para deter o crescimento da obesidade^37^ . A OMS preconiza medidas como taxação de alimentos ultraprocessados, subsídios aos alimentos saudáveis e proibição do marketing de alimentos para crianças^34 , 35^ . O México adotou uma lei taxando alimentos e bebidas ultraprocessados e com alto teor de açúcar em 2013, o que resultou na redução de 10% do consumo de refrigerantes e no aumento do consumo de água em 15%^34^ . Na Austrália, estudo de avaliação de custo-efetividade de intervenções para a redução do consumo de sal, por exemplo, identificou que, embora os programas educativos sejam efetivos, as medidas governamentais obrigatórias estabelecendo limites para a utilização de sal pelas indústrias podem ser até vinte vezes mais eficazes^36^ .

Ainda sobre a redução dos fatores de risco para as DCNT, o Brasil é considerado um exemplo mundial na redução da prevalência do tabagismo^38 , 39^ por tomar medidas como a proibição da propaganda do tabaco, adotar a Convenção-Quadro para o Controle do Tabaco em 2006 e aprovar a Lei nº 12.546 de 2011 e o decreto presidencial de 2014 instituindo os ambientes livres do fumo, elevando a taxação dos cigarros, entre outras medidas^39^ .

Neste estudo, comparados aos não fumantes, os tabagistas apresentaram menor prevalência de excesso de peso. Estudos apontam a relação inversa entre o tabagismo e peso corporal, atribuída à atuação da nicotina, tanto pelo aumento da atividade adrenérgica e gasto energético, contribuindo para a redução do peso corporal, quanto pela liberação de dopamina e serotonina, que atuam no controle e regulação do apetite no nível do hipotálamo^40^ . Tabagistas apresentaram maior chance de uso abusivo de álcool comparados aos não tabagistas em ambos os grupos etários. Estudo realizado em Portugal com adultos (≥ 19 anos) revelou maior consumo pelos fumantes de todas as bebidas alcoólicas avaliadas (cerveja, vinho, *whisky* e conhaque). Além disso, adultos e idosos que referiram consumo abusivo de álcool apresentam maior chance de ter uma dieta inadequada, com menor consumo de hortaliças e frutas em ambos os sexos^41^ . Dados do *National Health and Nutrition Examination Survey* mostram associação significativa entre o consumo de bebidas alcoólicas e a pior qualidade da dieta em homens e mulheres adultos (≥ 20 anos)^42^ .

Entre as limitações deste estudo, deve-se considerar que todas as informações foram autorreferidas, o que pode resultar em possível viés de informação sobre comportamentos considerados adequados. Em relação à amostra, ela se restringiu à população com telefone fixo na residência, o que pode diminuir a participação das regiões Norte e Nordeste do país em razão das menores taxas de cobertura. No entanto, o uso de fatores de ponderação reduz as diferenças observadas nas populações com e sem telefone^7^ .

Dado o delineamento transversal do estudo, nas relações entre os pares de fatores de risco avaliados, um OR > 1, por exemplo, indica que os indivíduos que exibem um determinado comportamento foram mais propensos a apresentar, naquele momento, o outro fator de risco do que os que não estavam expostos ao primeiro fator. Quanto ao impacto das exposições e comportamentos sociais ao longo da vida, Stenholm et al.^23^ apontam que a presença de no mínimo dois fatores de risco entre indivíduos de 50 a 75 anos reduz sua expectativa de vida saudável em oito anos e sua expectativa de vida livre de doenças crônicas em seis anos. O presente estudo aponta demandas em relação às políticas sociais e práticas em saúde, como por ações de regulação pública de fatores de risco em função do elevado percentual de indivíduos com múltiplos riscos, ao mesmo tempo em que identifica subgrupos com maiores riscos para DCNT comparados aos que apresentam apenas um ou nenhum dos fatores estudados. Assim, evidencia a necessidade de adoção de estratégias para intervenções sobre os múltiplos comportamentos e não apenas direcionadas aos fatores individuais.

Também é necessário ressaltar que, para a promoção de hábitos de vida saudáveis, as políticas macrossociais, intersetoriais e regulatórias são mais efetivas^34 , 35^ . Cabe destacar o papel do Estado enquanto agente regulador também em setores externos à saúde, para a promoção de hábitos de vida saudáveis. Já no âmbito individual de atenção à saúde, deve-se considerar que existe uma elevada prevalência aglomeração de fatores de risco na população e que os comportamentos substitutivos levam à manutenção ou à ampliação desses percentuais, caso não haja uma abordagem ampliada ao conjunto dos fatores. Assim, a integralidade como um valor a ser sustentado deve estar presente no encontro, na conversa em que o profissional de saúde busca reconhecer, para além das demandas explícitas, as necessidades dos usuários^43^ . O olhar ampliado para os problemas de saúde é uma condição fundamental e necessária em todos os serviços de saúde, já que o Estado tem o dever de oferecer um “atendimento integral, com prioridade para as atividades preventivas, sem prejuízo dos serviços assistenciais”, conforme a Constituição Federal de 1988.
